# Computer Assisted Self Interviewing in a Sexual Health Clinic as Part of Routine Clinical Care; Impact on Service and Patient and Clinician Views

**DOI:** 10.1371/journal.pone.0018456

**Published:** 2011-03-31

**Authors:** Lenka A. Vodstrcil, Jane S. Hocking, Rosey Cummings, Marcus Y. Chen, Catriona S. Bradshaw, Tim R. H. Read, Jun K. Sze, Christopher K. Fairley

**Affiliations:** 1 Melbourne Sexual Health Centre, Alfred Hospital, Victoria, Australia; 2 Sexual Health Unit, Melbourne School of Population Health, The University of Melbourne, Victoria, Australia; 3 Key Centre for Women's Health, Melbourne School of Population Health, The University of Melbourne, Victoria, Australia; 4 Department of Epidemiology and Preventive Medicine, Monash University, Victoria, Australia; University of Sao Paulo, Brazil

## Abstract

**Background:**

Computer assisted self interviewing (CASI) has been used at the Melbourne Sexual Health Centre (MSHC) since 2008 for obtaining sexual history and identifying patients' risk factors for sexually transmitted infections (STIs). We aimed to evaluate the impact of CASI operating at MSHC.

**Methodology/Principal Findings:**

The proportion of patients who decline to answer questions using CASI was determined. We then compared consultation times and STI-testing rates during comparable CASI and non-CASI operating periods. Patients and staff completed anonymous questionnaires about their experience with CASI. 14,190 patients completed CASI during the audit period. Men were more likely than women to decline questions about the number of partners they had of the opposite sex (4.4% *v* 3.6%, p = 0.05) and same sex (8.9% *v* 0%, p<0.001). One third (34%) of HIV-positive men declined the number of partners they had and 11–17% declined questions about condom use. Women were more likely than men to decline to answer questions about condom use (2.9% *v* 2.3%, p = 0.05). There was no difference in the mean consultation times during CASI and non-CASI operating periods (p≥0.17). Only the proportion of women tested for chlamydia differed between the CASI and non-CASI period (84% *v* 88% respectively, p<0.01). 267 patients completed the survey about CASI. Most (72% men and 69% women) were comfortable using the computer and reported that all their answers were accurate (76% men and 71% women). Half preferred CASI but 18% would have preferred a clinician to have asked the questions. 39 clinicians completed the staff survey. Clinicians felt that for some STI risk factors (range 11%–44%), face-to-face questioning was more accurate than CASI. Only 5% were unsatisfied with CASI.

**Conclusions:**

We have demonstrated that CASI is acceptable to both patients and clinicians in a sexual health setting and does not adversely affect various measures of clinical output.

## Introduction

The prevalence of sexually transmitted infections (STIs) is closely related to the community's access to clinical services [1]. This was illustrated in the UK when under-funding of genitourinary medicine (GUM) clinics led to reduced access to services and increases in gonorrhea rates which subsequently stabilized and declined with improved access [2], [3]. However obtaining sufficient funding for sexual health services is difficult while health care costs are rising at twice the rate of inflation [4]. It is therefore important that sexual health services like other areas of medicine, strive to increase their clinical efficiency.

One method that has been studied in a number of small randomized clinical trials or observational studies is computer assisted self interviewing (CASI). It was our hope that CASI may lead to improved efficiency and reduced cost of our sexual health clinical service [5]–[9]. The five published studies on CASI in STI services have generally shown that the benefit of operating CASI is that it improves the accuracy of the clinical information [5]–[9]. However, only one of these studies investigated the effect of CASI on clinical outcomes and reported that CASI may have contributed to a reduction of HIV testing rates [8], thus demonstrating a potential weakness of its introduction.

Very few clinical STI services have CASI operating as part of routine clinical practice and no studies have been published on CASI in routine practice. The earlier studies were relatively small and therefore had limited statistical precision but also may be biased because their participants may not have been representative of an entire clinical service. The Melbourne Sexual Health Centre (MSHC) is an STI service that sees approximately 20,000 individuals a year and has had CASI operating as part of routine clinical practice since mid-2008. CASI was introduced in the clinic with the aim to collect a core sexual history from a patient before they saw a clinician, thereby resulting in increased efficiency and more thorough risk profiles being identified. CASI obtains answers to questions about a patient's gender of sexual partners, STI history, and sexual risk behavior (i.e. condom use and number of partners). The CASI questions were chosen because they were deemed to be part of the core sexual history [10], [11]. When a patient completes CASI on one of the laptop computers in the clinic waiting room the data is stored on the main clinic server and a summary is printed on a sticker which is placed in the patients file. This allowed for an audit of CASI records which could be compared to data entered manually into the server when CASI was not available.

In 2010 all the laptop computers that ran CASI were stolen which resulted in its temporary discontinuation. This provided an unexpected opportunity to use this period as a control period to evaluate the effect of CASI on clinical practice. To complement this evaluation we also undertook surveys of both staff and patients to provide a complete picture of the acceptability of CASI as part of routine clinical practice.

## Materials and Methods

This study involved three separate components: a retrospective audit, a survey of patients and a survey of clinicians using CASI. It was conducted at MSHC a large urban sexual health service that provides a walk-in clinic. Patients attending the centre initially see a triage nurse to determine if they need to be seen. The triage nurse then classifies them as either needing to see a doctor (complicated cases e.g. symptomatic for an STI) or they could be seen by either a doctor or nurse (uncomplicated cases e.g. asymptomatic screen). Only men who have sex with men (MSM) are able to make appointments for first visits without triage.

CASI has been available for use at MSHC since June 2008 except for a four month period in 2010 (January 26^th^ – May 25^th^) following a robbery when the 10 laptop computers were stolen. At MSHC all new and returning patients attending the centre were first directed towards the 10 computer terminals in the foyer where they registered their demographic details (patient assisted registration), which was available in English, Thai, Korean and Chinese at the time of the evaluation. When this is complete the computer prompts all new individuals and those who have not been seen at MSHC for more than three months to begin their sexual history (available in English only at time of evaluation). Patients not wishing to do CASI can close the CASI screen or walk away from the computer which automatically closes after no activity for 3 minutes. CASI questions focus on five main areas; gender of sexual partners, number of sexual partners, drug use and history of both sexual health related infections and previous HIV testing.

For every CASI question, patients have the option to record ‘decline to answer’. After completing the CASI questions, patients are assessed by the triage nurse (and subsequently a clinician) using their paper medical record that includes a sticker with the summarized CASI history. This sticker includes the number of questions the patient has ‘declined to answer’.

### CASI retrospective audit

#### Proportion of patients who declined questions

To determine the proportion of individual patients who declined to answer specific questions we undertook a retrospective analysis of computer records of all patients undertaking CASI for the first time during the operating period (11^th^ June 2008 – 30^th^ June 2010, excluding the period when the computers were stolen). If patients were eligible for CASI twice between June 2008 and June 2010 only their first visit was included in the analysis. Sex workers were excluded from the analysis because they attend more often than every three months and because sex workers are not easily compared from one country to another and hence limit the generalizability of the study findings. In order to calculate the proportion that declined to answer each question on CASI, we divided the number who declined to answer each specific question into the number who were asked that question.

### CASI compared with non-CASI periods

To determine if CASI influenced clinical practice we compared components of clinical practice during three specific 12-week time periods: one period when CASI was operating (1^st^ February – 30^th^ April 2009) and two similar time periods when CASI was not operating (1^st^ February – 30^th^ April 2008 before CASI was instituted and 1^st^ February – 30^th^ April 2010 when CASI ceased to operate). By including the same period (end of Summer to mid-Autumn) we removed seasonal variability from our analysis. For the CASI and non-CASI periods we used data from all patients who would be eligible to use CASI (new patients or those not seen for more than three months excluding sex workers) in each of the three 12-week periods. Only a patient's first visit within each specific 12-week period was included in the analysis.

#### Consultation duration in CASI compared with non-CASI periods

The time taken for a consultation was recorded by the center's computer. The data analysis for the duration of consultations included only consultations of clinicians who worked in all three of the time periods. If a clinician only worked in one or two of the time periods, their data was excluded to avoid the individual characteristics of these clinicians influencing the consult duration. This analysis also excluded patients who were seen in less than 5 minutes or who took more than 90 minutes as these data are likely to be errors associated with clinicians not recording start and finish times correctly.

#### Uptake to STI testing in CASI compared with non-CASI periods

To determine if CASI influenced STI testing rates we compared STI testing during the CASI and non-CASI periods. It was expected that there be no differences in the uptake to STI or HIV testing in patients who completed or did not complete CASI. We looked at chlamydia tests, HIV tests for all patients and anal swabs taken from MSM for either chlamydia and/or gonorrhea. The data analysis was restricted to patients who saw a clinician who was working during each of the three periods.

### Patient questionnaire

To determine the patient's views on CASI we used one page self-completed, anonymous questionnaires containing nine questions that were given out after patients were assessed by the triage nurse for a one week period (31^st^ May – 4^th^ June 2010). Questions included demographics such as gender, age, gender of sexual partners, and whether the patient was a new patient to the clinic or a returning patient. Patients were also asked to rate the ease of computer use, describe the accuracy of their answers and rate how comfortable they were answering the questions on a computer. A final question asked whether patients would prefer using the computer for a self-interview or being asked the questions face-to-face by the treating clinician. Patients also had an opportunity to provide an open-ended comment about the computers.

### Clinician, triage nurse and administration staff questionnaires

The staff survey was conducted using an internet survey program, SurveyMonkey™ (www.surveymonkey.com). All MSHC staff members were provided with a link to the online questionnaire together with a letter explaining that their answers would remain confidential and anonymous.

Clinicians were asked whether in their view CASI or face-to-face interviewing was: better for obtaining accurate information about gender and number of sexual partners, condom use, injecting drug use, and last HIV test; if they would recommend CASI to another clinic; how useful CASI was and how it affects the building of rapport with patients; and whether or not they were satisfied with CASI operation. Triage nurses had additional questions about how CASI affects the triage process. Administration staff were asked about their opinions on how CASI affects patient flow in the clinic and how often patients ask for help. Most questions provided an opportunity to expand on answers.

### Statistical Analysis

#### Patient response rate and staff and patient questionnaires

Chi squared analysis was used for exploring differences in patient response rate between gender and for analyzing associations between categorical variables in staff and patient questionnaires.

#### Consultation duration and STI testing

Linear regression analysis was used to determine differences in consultation time between CASI and non-CASI periods, adjusting for potential intra cluster correlation among individual clinicians. Logistic regression was conducted to evaluate whether the proportion of patients who has an STI test varied between CASI and non-CASI periods adjusting for potential intra cluster correlation among individual clinicians.

### Ethics Statement

Ethical approval was obtained from the Alfred Hospital Research Ethics Committee who approved written consent not being obtained to preserve the anonymous nature of the questionnaire. The ethics committee also approved no consent being obtained for the retrospective analysis of existing data because it involved no risk to confidentiality.

## Results

### CASI retrospective audit

**Figure 1:**
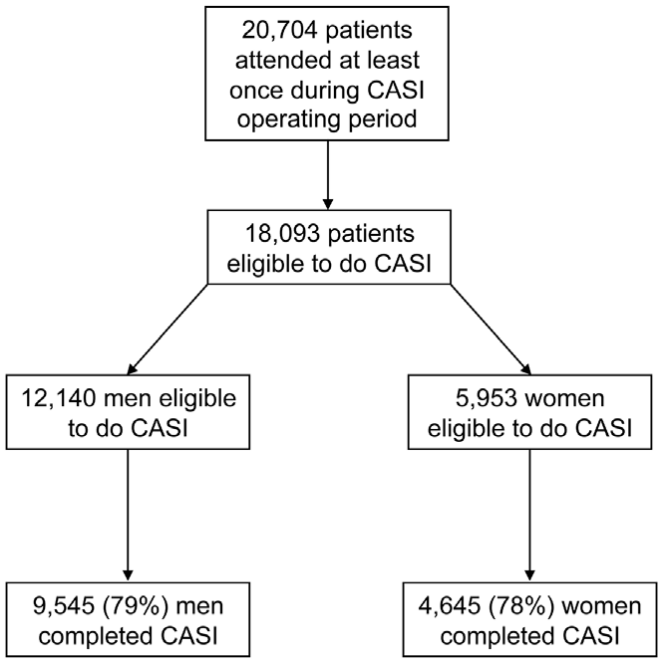
Diagram of patients attending the Melbourne Sexual Health Centre and those that were eligible and completed computer assisted self interviewing (CASI).

There were 20,704 individual patients who attended the centre at least once during the CASI operating period (11^th^ June 2008 to 30^th^ June 2010, excluding the period from 26^th^ January 2010 to 24^th^ May 2010 when CASI was not in operation) (Figure 1). Of these individual patients, 18,093 (12,140 men and 5,953 women) were eligible to do CASI on their first visit during this period (sex workers excluded). Of the eligible patients, 9,545 (79%) men and 4,645 (78%) women completed CASI.

#### Proportion of patient who declined questions

The proportions of men and women who declined different questions in CASI ranged from 0–8.9% (Table 1). There were significant differences in the proportion of questions that were declined by men and women. Men were significantly more likely than women to decline questions about how many casual sexual partners (CSPs) of the opposite sex they had had in the last 12 months (p = 0.05), and how many CSPs of the same sex they had had in the last 12 months (p<0.001). Women were significantly more likely than men to decline to answer questions about condom use with CSPs of the opposite sex in the last 12 months (p = 0.05). There was no difference between men and women in reporting condom use with regular sexual partners of the opposite sex.

**Table 1:** Number of male and female patients that declined to answer specific questions during CASI.

	*Male patients*			*Female patients*			
*Questions asked by CASI*	n	N	%	n	N	%	*p-value* a
Have you been diagnosed with an STI	72	9545	0.8	44	4645	0.9	0.27
When was your last HIV test	51	9337	0.5	18	4569	0.4	0.28
Have you had a Pap smear	n.a	n.a		0	4645	0.0	
Was your Pap smear normal	n.a	n.a		7	3314	0.2	
Are you pregnant	n.a	n.a		17	4574	0.4	
Are you trying to conceive	n.a	n.a		6	4058	0.1	
What are your contraception methods	n.a	n.a		115	2965	3.9	
Do you Inject drugs	93	9501	1.0	42	4574	0.9	0.80
Have you had a Hepatitis C test	1	353	0.3	0	218	0.0	0.81
***SEXUAL BEHAVIOUR***							
Have you had sex in the last 12 mths	66	9501	0.7	26	4576	0.6	0.45
***Sex with opposite sex***							
Sex with opposite sex last 12 mths	24	9158	0.3	7	4393	0.2	0.33
Opposite sex RSP	77	6315	1.2	51	4287	1.2	0.96
Opposite sex RSP condom use	52	3395	1.5	36	2414	1.5	0.99
Last 12 mth opposite sex number of CSP	277	6315	4.4	155	4287	3.6	*0.05*
Last 12 mth opposite sex condom use	128	5638	2.3	110	3735	2.9	*0.05*
***Sex with same sex***							
Sex with same sex last 12 mths	72	9158	0.8	26	4393	0.6	0.25
Same sex RSP	50	3211	1.6	2	333	0.6	0.25
Male RSP condom use RAS	33	1326	2.5	n.a	n.a		
Male RSP condom use IAS	36	1326	2.7	n.a	n.a		
Last 12 mth same sex number of CSP	285	3211	8.9	0	333	0.0	*<0.001*
Last 12 mth male CSP anal sex	47	3067	1.5	n.a	n.a		
Last 12 mth male CSP condom use RAS	32	2442	1.3	n.a	n.a		
Last 12 mth male CSP condom use IAS	47	2442	1.9	n.a	n.a		
Unprotected anal sex since last HIV test	70	2706	2.6	n.a	n.a		

a Chi square test for differences in the proportion who declined by gender; n  =  number of patients who declined the question asked, N  =  total number of patients asked the question, RSP  =  regular sexual partner, CSP  =  casual sexual partner, RAS  =  receptive anal sex, IAS  =  insertive anal sex, mth  =  month.

When answering questions on CASI about sexual activity, HIV-positive MSM were significantly more likely than HIV-negative MSM to decline questions about the number of CSPs, anal sex with CSPs and condom use with CSPs compared with HIV-negative men (p<0.002) (Table 2).

**Table 2:** Number of HIV-positive and HIV-negative MSM that declined to answer specific questions about their sexual history during CASI.

	*HIV-positive*			*HIV-negative*			
*Questions asked by CASI*	n	N	%	n	N	%	*p-value* a
Do you have a male RSP	1	41	2.4	51	3172	1.6	0.84
RSP condom use RAS	0	17	0.0	34	1310	2.6	0.59
RSP condom use IAS	0	17	0.0	36	1310	2.8	0.56
How many CSPs have you had in the last 12 mths	14	41	34.2	272	3030	9.0	*<0.001*
Have you had anal sex with CSPs	4	39	10.3	44	3030	1.5	*<0.001*
CSP condom use RAS	4	34	11.3	56	2489	2.3	*0.002*
CSP condom use IAS	6	34	17.7	73	2489	2.9	*<0.001*

a Chi square test for differences between HIV-positive and HIV-negative MSM; n  =  of patients who declined the question asked, N  =  number of patients asked the question, RSP  =  regular sexual partner, CSP  =  casual sexual partner, RAS  =  receptive anal sex, IAS  =  insertive anal sex.

### CASI v non-CASI periods

#### Consultation times with CASI compared with non-CASI periods

The mean consultation times for the CASI and non-CASI period stratified by the type of clinician (doctors or nurses) and the type of consultation (complicated, uncomplicated or appointment) are demonstrated in Table 3. There were no significant differences in the mean consultation times between the CASI and non-CASI period (p≥0.17).

**Table 3:** Mean consultation times of doctors and nurses seeing patients who have appointments or have been triaged in to the clinic as ‘complicated’ or ‘uncomplicated’ patients in CASI (2009) and non-CASI (2008 and 2010) periods.

		*CASI*		*non-CASI*		
	*Consult type*	n	mean (95% CI) (minutes)	n	mean (95% CI) (minutes)	*p-value* a
*Doctor*	appointment	88	42.7 (38.4–46.9)	185	40.6 (36.3–44.9)	0.41
	complicated	901	37.6 (33.5–41.6)	1862	36.4 (32.6–40.2)	0.31
	uncomplicated	439	26.7 (23.3–30.1)	920	26.2 (23.6–30.6)	0.70
*Nurse*	appointment	61	27.8 (24.4–31.2)	181	27.1 (23.6–30.6)	0.69
	uncomplicated	399	25.0 (19.1–31.0)	1017	27.2 (24.2–30.1)	0.17

a Linear regression analysis for difference in consult time between CASI and non-CASI periods adjusting for potential intra cluster correlation from individual clinicians. n  =  total number of consults. Data was for 13 nurses and 17 doctors who saw patients in all of the three time periods.

#### Uptake to STI testing with CASI compared with non-CASI periods

To determine if there were any differences in STI testing rates between CASI and non-CASI periods, an analysis of the uptake to STI testing was performed (Table 4). Only the proportion of women tested for chlamydia was different in the CASI period compared with the non-CASI period (p<0.001, 84% *v* 88% respectively).

**Table 4:** Number of chlamydia (from any site), HIV tests and anal swabs ordered in CASI (2009) and non-CASI (2008 and 2010) periods.

		*CASI*			*non-CASI*			
	*Test*	n	N	%	n	N	%	*p-value* a
*Heterosexual men*	chlamydia	607	748	81	1330	1643	81	0.85
	HIV	323	715	45	687	1551	44	0.61
*MSM*	chlamydia	408	441	93	981	1045	94	0.40
	HIV	343	436	79	849	1041	82	0.23
	anal swab	369	441	84	842	1045	81	0.38
*Female*	chlamydia	645	767	84	1525	1735	88	*<0.001*
	HIV	330	751	44	817	1686	48	0.18

a Logistic regression for differences in testing rates between CASI and non-CASI periods adjusting for potential intra cluster correlation from individual clinicians. n  =  number of patients tested; N  =  number of patients seen during each period. MSM  =  men who have sex with men.

### Patient questionnaire

During the survey week, 306 patients answered CASI and of these 267 (87%) patients completed the questionnaire after being triaged. Only 266 specified their gender and were included in the analysis (Table 5). Most respondents (72% men and 69% women) were comfortable using the computer and most reported (76% men and 71% women) that their answers to all questions were accurate. Women (19%) were more likely than men (7%) to report difficulty in using the computer (p = 0.02). About half of patients preferred to use CASI but a significant minority (18%) would have preferred a clinician to have asked the questions in person.

**Table 5:** Patient questionnaire separated for males and females.

	*Male*			*female*			
*Patient questionnaire questions*	N	n	%	N	n	%	*p-value* a
*Gender of sexual partners*							
female	175	110	63	79	2	23	
male		58	33		68	86	
both male and female		7	4		9	11	
*Patient age*							
less than 25	180	47	26	85	37	44	
greater than or equal to 25		133	74		48	56	0.004
*New or returning patient*							
new	180	83	46	84	44	52	
returning		97	54		40	48	0.34
*How did you find using the computer?*							
very easy, easy	180	149	83	86	61	71	
neither easy nor difficult		18	10		9	10	
difficult, very difficult		13	7		16	19	0.02
*How comfortable did you feel using the computer?*							
very comfortable, comfortable	180	130	72	86	59	69	
neither comfortable nor uncomfortable		32	18		15	17	
uncomfortable, very uncomfortable		18	10		12	14	0.63
*How accurate were your answers to the computer questions?*							
All were accurate	180	136	76	84	60	71	
Some were accurate		42	23		24	29	
Not many, none were accurate		2	1				0.64
*Would you prefer a computer or clinician for answering the questions?*							
Strongly prefer or prefer computer	180	87	48	85	42	49	
Don't mind computer or clinician		64	36		23	27	
Strongly prefer or prefer clinician		29	16		20	24	0.22

a Chi square test for differences in responses between genders; N  =  number of respondents of each sex for each question; n  =  number of patients who chose the option. One patient was excluded because they did not complete their gender.

There were 84 comments made by respondents in the free text box; 52 of the comments concerned technical difficulties mainly to do with the sensitivity of the touch screens of the computers on which CASI operates in the reception area. Twelve had positive feedback including that it was a ‘good innovative system which provides pre-consultation information’, that it was an ‘easier and faster method’ and that it was very ‘clear and quick’ to use. Four patients had general suggestions to improve clarity of questions. Fourteen of the patients who received the questionnaire reported that they found CASI either repetitive, confusing, over complicated or intrusive.

### Clinician questionnaire

During the survey period, 19 of 22 doctors and 20 of 22 nurses who were currently employed and had worked during both CASI and non-CASI periods completed the staff survey. There was no statistical difference (p>0.14) in the responses of nurses and doctors to any questions and therefore responses were combined in Table 6. A significant minority (range 11%–44%) of clinicians felt that when identifying STI risk factors face-to-face questioning was more accurate than CASI. For example, 44% of clinicians believed face-to-face was more accurate for obtaining answers about condom use.

**Table 6:** Clinician questionnaire answers.

*Clinician questionnaire questions* a	N	%	95% CI
*Do you think that CASI or face-to-face questioning provides more accurate answers about the following?*			
** Gender of partners**			
face-to-face	12	31	18, 48
Same	16	41	26, 58
CASI	11	28	16, 45
cannot make this assessment	0	0	-
** Number of partners**			
face-to-face	15	39	24, 55
Same	11	28	16, 45
CASI	11	28	16, 45
cannot make this assessment	2	5	1, 19
** Condom use**			
face-to-face	17	44	12, 40
Same	13	33	20, 50
CASI	6	15	6, 31
cannot make this assessment	3	8	2, 22
** Injecting drug use**			
face-to-face	4	11	4, 26
Same	16	43	27, 60
CASI	12	32	19, 50
cannot make this assessment	5	14	5, 30
** Last HIV test**			
face-to-face	15	39	25, 57
Same	15	39	25, 57
CASI	3	8	2, 22
cannot make this assessment	5	3	5, 29
*How does CASI affect your ability to build rapport?*			
Better	18	47	31, 64
Unchanged	18	47	31, 64
Worse	2	5	1, 19
*How has CASI affected the quality of your consult?*			
Better	24	63	46, 78
Unchanged	13	34	20, 51
Worse	1	3	0, 15
*How has CASI affected your consult time?*			
Quicker	24	63	46, 78
Unchanged	13	34	20, 51
Longer	1	3	0, 15
*How would you recommend CASI to another clinic?*			
Recommend	34	89	74, 97
neither recommend or not recommend	3	8	2, 22
not recommend	1	3	0, 15
*How satisfied are you with CASI?*			
Satisfied	30	84	68, 93
neither satisfied nor unsatisfied	4	11	3, 26
Unsatisfied	2	5	0, 19

a There was no statistical difference (p>0.14) between responses by doctors or nurses so answers were combined for both. N  =  total number who answered each response of the question; face-to-face  =  questions asked by a clinician; CASI  =  computer assisted self interviewing.

Clinicians overwhelmingly supported CASI with 84% of clinicians reporting they were satisfied with it and only one felt that CASI adversely affected their consultations. 63% felt it improved the quality of their consults and made consults quicker and 89% would recommend CASI being implemented at another clinic. Examples of some of the text responses provided are demonstrated in Table 7.

**Table 7:** Examples of comments made by clinicians about CASI operating at MSHC.

	*Examples of responses and free text comments*			
	*Nurses*		*Doctors*	
*Question*	*response*	*free text*	*response*	*free text*
*Do you think that CASI or face-to-face questioning provides more accurate answers to questions about condom use?*	Face to face more accurate	This question usually needs more investigation face to face as there are different scenarios/variations of condom use, especially with regular sexual partners.	Face to face more accurate	I frequently find that a condom hasn't been used when a patient has said they are always used.
	CASI more accurate	But still have to check [with the patient] as it doesn't allow for broken condoms.	Face to face more accurate	When asking about how a condom was used, face to face will reveal that the condom was put on 1/2 way through sex.
*Do you think that CASI or face-to-face questioning provides more accurate answers to questions about drug use?*	Neither CASI or face to face more accurate	Sometimes alcohol or recreation drug use comes out later in the consultation, especially when you are exploring why the unsafe sex occurred.	CASI more accurate	I am not very good about always asking about drug and alcohol use - but I do when in relation to unprotected sexual intercourse in MSM.
	Face to face more accurate	This is a question [about drug use] that I think patients are more likely to decline to answer on CASI.	Neither CASI or face to face more accurate	Relatively similar; however, this is a good question for CASI, as the clinician may not always ask this question.
*Which of the following options best describes how CASI has affected your ability to establish rapport with patients?*	Better	Provides better opportunities of exploring other more pertinent history taking - such as relationships, support structures, drug and alcohol use etc.	Unchanged	I sometimes wonder if it short cuts the process of making rapport, but it does get to the risk factors quicker.
	Better	The patients appear more comfortable when they enter consultation. They have already spent time considering their questions and risk.	Better	It "breaks the ice" regarding the confronting questions of casual sexual partner/same-sex partner, numbers of partners etc.
*Has CASI affected the quality of your consultations?*	Significantly better	The patient has thought about there sexual behavior also they have an idea how/what we may ask further reduces embarrassment.	Worse	Generally get less of an idea about the social and temporal situation of sex partners, and I feel less inclined to delve.
	Unchanged	Overall I don't think CASI has changed the quality of my consultations, it's just a different way of gathering information.	Better	CASI remembers to ask things I might forget, especially things that are less commonly important, but still can be important.
*Which of the following options best describes how CASI has effected the time taken for you to complete your consultations?*	Not changed	Even though a small amount of time might be saved not asking all the questions which are now covered by CASI, I still talk about the issues that have brought the patient to MSHC and can focus on them more.	Reduced time	I suspect it saves time. Sometimes it costs time clearing up CASI-confusion, where a partner has been double-entered [as a regular and casual sexual partner], but this is not frequent.
	Significantly reduced time	So much of the history is already taken care of in CASI so it saves time in asking these questions and also allows more time to explore other issues.	Not changed	In some consultations it has set the agenda and in others has created some level of confusion that needs resolution.

CASI  =  computer assisted self interviewing, MSM  =  men who have sex with men.

### Triage questionnaire

All (n = 16) nurses who worked in triage and responded to the online questionnaire were satisfied with CASI operating at MSHC and 88% (14) thought CASI improved the triage process.

### Administration questionnaire

Four of six administration staff members believed that CASI improved flow of patients through the registration process whereas the other two thought CASI made flow worse. Most administration staff said they had to help patients with the computer ‘often’ or ‘sometimes’ mainly with technical issues the patient faced.

## Discussion

This is the first evaluation of CASI in operation in routine practice in a sexual health. We found that patients declined to answer questions infrequently, most were comfortable and preferred using CASI, and most claimed to answer questions accurately. CASI had little influence on the duration of consultations. There is the possibility that it could have reduced chlamydia testing in women, although in this observational analysis, it is perhaps most plausible that other unmeasured differences accounted for this finding. In general, CASI was not expected to change uptake to STI testing as patients report risk behavior to both CASI and in face-to-face interviewing. Staff were satisfied with CASI but did not always think CASI was better for obtaining accurate information about sexual risk. They did think that it improved clinical care in terms of increased quality of consults and shorter consult times. These findings from a large evaluation, in addition to the smaller analytical studies generally support the introduction of CASI into clinical STI services [5]–[9]. The true value of CASI is most likely to be realized when it is integrated with further innovations in clinical care such as the development of express clinical services, although CASI operating alone allows for the development of a detailed behavioral surveillance system [12], [13].

Relatively few patients declined to answer questions when CASI was operating as part of routine care. The exception to this was HIV-positive men where over one third declined to answer questions about the number of recent male sexual partners. In addition, about one in 20 men and women declined to answer questions about the number of opposite sex partners. Our observation that overall few patients declined to answer the majority of questions must be tempered by the fact that about 20% of patients did not undertake CASI in our study. Unfortunately we do not have information on why patients chose not to undertake CASI but reasons may have included: the CASI terminals being occupied when the clinic was busy; there was insufficient time just before closing time; or because CASI was only available in English throughout this evaluation period.

We did not find any objective difference in consultation times during CASI and non-CASI periods despite the majority of clinicians subjectively feeling that CASI shortened their consultations. The objective analysis involved a large number of consultations, was corrected for the complexity of the patients seen and adjusted for the clinician seeing them. Our conclusion from this data is that if CASI does shorten consultations, it is not by a clinically important difference. It is possible that if time was saved using CASI it may have been used by clinicians in other parts of the consultation that could explain the 63% of clinicians who felt that the quality of the consultation was improved.

In general CASI had no effect on the use of diagnostic tests with the possible exception of reducing the number of chlamydia tests ordered for females. However, the absolute difference in chlamydia testing rates was small (84% tested during CASI periods vs. 88% for non-CASI periods) and this difference may have been due to other unmeasured factors. Given that the CASI histories generally identify a higher risk among heterosexual women, it is difficult to postulate a plausible rationale for why chlamydia testing rates would be lower in this group [9]. In the UK, a randomized controlled trial reported lower HIV testing rates in patients who entered information in CASI compared with patients who were interviewed with the traditional pen and paper interview [8]. We observed no significant differences in HIV testing rates in our study.

In the current study of CASI as part of routine care, a somewhat lower proportion of patients were comfortable or very comfortable using CASI (71%) than found in a randomized study at our centre (86%) [9]. Similarly in the current study a lower proportion of patients found using CASI easy or very easy (79%) than found in the randomized study (86%) [9]. This small but important difference may have been because in the randomized study patients were required to initially consent to participate which may have resulted in a sample that were more comfortable with using computers [9].

The current study highlighted the complexity of the accuracy of the answers to sensitive questions about sexual risk. In our patient survey only about 75% of patients reported that their answers to the questions were *all* accurate, although only 1% reported that few were accurate. It is important to note that we did not aim to compare the accuracy of answers between CASI and face-to-face interviews. We obtained opinions from clinicians and patients as to how accurate they think responses to CASI are. Therefore, the responses we received may be biased if patients report that their answers are accurate if it is socially desirable to do so and if clinicians make assumptions about the accuracy of CASI obtained information. Given that previous research has shown that CASI obtains more accurate answers to socially desirable questions than clinicians [5]–[9] it is likely that less than 75% of patients answer all questions accurately to clinicians. However in the current study clinicians felt that they could obtain more accurate answers than CASI. The clinician's view that they can obtain more accurate answers may in part be explained by the complexity of some questions that require detailed clarification (e.g. condom use) although this cannot explain their views in relation to simple questions about the sex of partners.

There are a number of limitations to our study. Firstly, it was an observational study that was subject to the biases associated with such studies. For example there may have been systematic differences between the three time periods that may have either resulted in significant findings that were attributed to the influence of CASI or alternatively disguised real effects of CASI. We lessened this possibility by standardizing the time of year, clinician and type of consult but other unmeasured factors cannot be excluded. We note that all real life evaluations of interventions suffer from this limitation. Secondly, a significant minority of patients during the CASI period did not undertake CASI but we would make the point that our evaluation was one of a clinic with and without CASI operating and not a comparison of patients who did and did not use CASI. Another point that requires consideration is that most patients know that their responses to CASI will eventually be read by a triage nurse or clinician. Thus their responses will not remain anonymous during their consult. This point does not explain why studies have reported that CASI obtains more accurate answers to socially undesirable questions [5]–[9], but might explain why we reported no differences in uptake to STI testing, and why clinicians believe they obtain more accurate answers to some risk behavior questions.

This study had a number of important strengths. Firstly, it represented a large number of patients undergoing CASI as part of routine clinical care. Secondly, we used two control periods on either side of the CASI period to hopefully minimize other factors, such as temporal changes that may have been different between CASI and non-CASI periods. Thirdly, the periods of time we analyzed were the same each year allowing us to remove seasonal variability from our analysis.

Having established that CASI can operate successfully as part of routine clinical practice it is now important to undertake research on how CASI can be best used to develop express clinical services, that both improve the efficiency and effectiveness of clinical care with the important population health consequences of improved STI rates [14], [15]. For example, while it may be safe to operate an express clinical service for low risk heterosexuals, using such a service for MSM, who are at significant risk of HIV may forego the opportunities to encourage condom use that might occur when a clinician and patient talk directly [14]. Some of these concerns may be circumvented by further innovative developments with computer counseling but one could argue that the introduction of these developments should be carefully evaluated like all important health interventions before being introduced [16], [17].
